# Coordinating mitochondrial translation with assembly of the OXPHOS complexes

**DOI:** 10.1093/hmg/ddae025

**Published:** 2024-05-23

**Authors:** Laura S Kremer, Peter Rehling

**Affiliations:** Department of Cellular Biochemistry, University Medical Center Göttingen, Humboldtallee 23, Göttingen 37073, Germany; Department of Cellular Biochemistry, University Medical Center Göttingen, Humboldtallee 23, Göttingen 37073, Germany; Cluster of Excellence “Multiscale Bioimaging: from Molecular Machines to Networks of Excitable Cells” (MBExC), University of Göttingen, Robert-Koch-Str. 40, Göttingen 37075, Germany; Fraunhofer Institute for Translational Medicine and Pharmacology, Translational Neuroinflammation and Automated Microscopy, Robert-Koch-Str. 40, Göttingen 37075, Germany; Max Planck Institute for Multidisciplinary Science, Am Faßberg 11, Göttingen 37077, Germany

**Keywords:** Mitochondria, Gene expression, Oxidative phosphorylation (OXPHOS), Translation, OXPHOS assembly, Coordination of translation and assembly

## Abstract

The mitochondrial oxidative phosphorylation (OXPHOS) system produces the majority of energy required by cells. Given the mitochondrion’s endosymbiotic origin, the OXPHOS machinery is still under dual genetic control where most OXPHOS subunits are encoded by the nuclear DNA and imported into mitochondria, while a small subset is encoded on the mitochondrion’s own genome, the mitochondrial DNA (mtDNA). The nuclear and mtDNA encoded subunits must be expressed and assembled in a highly orchestrated fashion to form a functional OXPHOS system and meanwhile prevent the generation of any harmful assembly intermediates. While several mechanisms have evolved in eukaryotes to achieve such a coordinated expression, this review will focus on how the translation of mtDNA encoded OXPHOS subunits is tailored to OXPHOS assembly.

## Introduction

Mitochondria are double-membrane enclosed organelles that exist in almost all eukaryotic cells. They are known as the powerhouse of the cell due to their crucial function in energy conversion carried out by the oxidative phosphorylation (OXPHOS) system. The mammalian OXPHOS system consists of five multimeric protein complexes known as OXPHOS complexes I to V. Complexes I to IV constitute the electron transport chain (ETC). They utilize the energy which is released upon transferring electrons from reducing equivalents derived from nutrients onto oxygen to power the translocation of protons across the inner mitochondrial membrane, thereby creating an electrochemical proton gradient. This gradient is then harnessed by complex V, also known as F_1_F_o_-ATP synthase, to produce ATP. Reminiscent of the mitochondrion’s endosymbiotic origin, the OXPHOS machinery is under dual genetic control: most of the OXPHOS subunits along with other proteins important for mitochondrial function are encoded by the nuclear DNA, translated by cytosolic ribosomes and imported into mitochondria. However, a small subset of core structural subunits of the OXPHOS machinery along with rRNAs and tRNAs required for their translation is encoded by the mitochondrial DNA (mtDNA). Eventually, the nuclear and mtDNA encoded subunits must come together to create a functional OXPHOS system. Importantly, this must occur in a highly coordinated fashion, as misbalance between the nuclear and mtDNA encoded subunits can result in proteotoxic stress or the generation of reactive oxygen species (ROS), eventually compromising cellular and organismal health [1–3]. This task is however not trivial, as the principles of nuclear and mitochondrial gene expression largely differ. Nuclear gene expression, especially of genes encoding proteins acting together in complexes, is largely controlled by transcription initiation at individual promoters [4–6]. In contrast to the nuclear DNA, mtDNA is transcribed as nearly genome-length polycistronic transcripts. Regulation of individual transcripts by transcription initiation is therefore not possible. Yet, the different steady-state levels of mitochondrial encoded mRNAs as well proteins, alongside with the different stoichiometries of the OXPHOS complexes, points to regulatory mechanisms at the post-transcriptional level [7–9]. This review will focus on the aspect of translational regulation of mitochondrial gene expression and how it is tailored to and by the assembly process of the OXPHOS complexes.

## Mitochondrial translation

Despite their bacterial origin, mitochondria have a unique gene expression machinery, including their mRNA architecture, their genetic code, and their translation apparatus. Mitochondrial mRNAs (mt-mRNAs) lack 5′-caps, which are important for regulation of translation initiation of nuclear genes, as well as Shine-Dalgarno sequences, controlling translation initiation in the bacterial system [10]. It is therefore still unknown how the START codon is recognized and translation is initiated. Mitochondrial translation is furthermore carried out by ribosomes which are specific to mitochondria. The mitochondrial ribosome is extremely protein-rich compared to its bacterial and cytosolic counterparts and only about half of the mitochondrial ribosomal proteins have bacterial homologs [11–14]. The baker’s yeast mitochondrial ribosome, for example, consists of 2 rRNAs, namely the15S rRNA of the small subunit (mtSSU) and the 21S rRNA of the large subunit (mtLSU), and 73 proteins [15]. The human 55S mitochondrial ribosome contains the 12S mtSSU rRNA and the 16S mtLSU rRNA and 82 proteins [14]. According to their specialized make-up, mitochondrial ribosomes also use a unique set of translation initiation, elongation, and termination factors, culminating in a translation cycle that is substantially different from the bacterial and cytosolic ones [16, 17]. Furthermore, mitochondrial ribosomes are tightly associated with the inner mitochondrial membrane. This facilitates the efficient cotranslational insertion of the highly hydrophobic mtDNA encoded proteins and prevents protein aggregation during transport. In yeast, the anchoring of the mitochondrial ribosome can be mediated by several contacts, including the mitochondrial ribosome receptor Mba1 and the membrane protein Mdm38, which bring the polypeptide tunnel in close vicinity to the insertase Oxa1 [18, 19]. In humans, the Mba1 homolog mL45, which is an integral part of the mitochondrial ribosome, together with two other mitochondrial ribosomal proteins establishes the contact with the OXA1L insertase [20].

## OXPHOS assembly

To form functional OXPHOS complexes, the mtDNA encoded subunits must eventually be assembled together with the nuclear encoded subunits in a concerted manner. OXPHOS assembly requires a large repertoire of assembly factors assisting the correct folding of subunits, the addition of cofactors, the stabilization of assembly intermediates, as well as the incorporation of OXPHOS subunits into the respective complexes. The required factors and assembly lines largely differ between the individual complexes and are reviewed in its entirety elsewhere [21–23]. As an example, we will sketch the assembly of complex IV.

Mammalian complex IV consists of 14 subunits: the three mtDNA-encoded catalytic subunits COX1, COX2, and COX3 and 11 nuclear encoded subunits important for the stabilization and regulation of the catalytic core. The proper assembly of these subunits into a functional complex IV requires more than 30 assembly factors. Amongst others, these are crucial for the correct insertion of heme and copper into the electron-transferring metal centers of COX1 and COX2, thereby preventing the generation of harmful radicals such as ROS by uncontrolled electron flux. The assembly of complex IV proceeds through the subsequent association of modular subassemblies. Initially, the COX1 module, whose assembly is facilitated through the MITRAC (Mitochondrial Translation Regulation Assembly intermediate of Cytochrome c oxidase) complex, comes together with the COX4-COX5A module [24, 25]. Successively, the COX2 module and COX3 module follow. Complex IV assembly is completed with NDUFA4 addition [21].

## Coordinated mitochondrial translation and OXPHOS assembly in yeast

**Figure 1:**
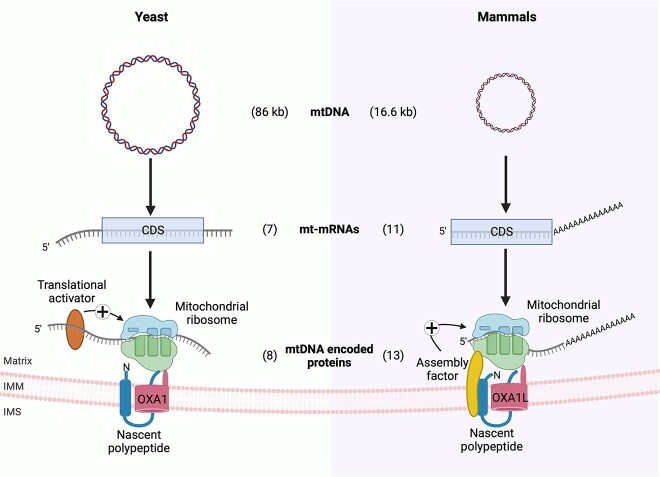
Mitochondrial gene expression in yeast and mammals. Transcription of the 86 kb yeast mitochondrial DNA (mtDNA) generates 7 mitochondrial mRNAs (mt-mRNAs) that contain long 5′ untranslated regions (UTRs) but no polyA-tails. The mt-mRNAs are translated by mitochondrial ribosomes tethered to the inner mitochondrial membrane (IMM) to produce 8 mtDNA encoded proteins. Translation of these proteins is regulated by translational activators, which can take part in feedback loops between OXPHOS assembly and translation. The mammalian mtDNA is 16.6 kb in size and its transcription produces 11 mt-mRNAs. Importantly, mammalian mt-mRNAs contain no or only very short 5′ UTRs. Instead, they possess polyA-tails. Translation produces 13 mtDNA encoded proteins. The translation of some of these proteins has been shown to be controlled by early assembly intermediates. Abbreviations: CDS, coding sequence; IMS, intermembrane space. The figure has been created using BioRender.com.

Much of our knowledge on how mitochondrial translation and OXPHOS assembly are coupled is derived from studies in *Saccharomyces cerevisiae* [26]. Its 86 kb mtDNA is transcribed as several polycistrones, which are further processed to generate seven mature mRNAs, encoding eight proteins: one complex III subunit, three complex IV subunits, three complex V subunits, and one ribosomal small subunit [12, 27, 28] (Fig. 1). In contrast to the mammalian system, yeast mtDNA does not encode any complex I subunit, as yeast possesses single subunit NADH-quinone oxidoreductases instead of a multimeric complex I [29, 30]. Furthermore, no complex II subunit is encoded on the yeast mtDNA, as is the case in mammals. Peculiar to the yeast system, yeast mt-mRNAs do not have polyA-tails, but instead possess 3′ untranslated regions (UTRs) that are important for 3′ end processing and mt-mRNA stability [31–33]. Additionally, they contain long 5′ UTRs which immediately suggests an important regulatory function. Indeed, specific proteins binding the 5′ UTR and thereby regulating translation, so called translational activators, have been identified for each yeast mt-mRNA [11]. The full molecular picture of how all of these translational activators exert their precise function is still emerging. For some translational activators it has been shown that the mere abundance of the activator is responsible for modulating the translation of its client mt-mRNA [16]. Other translational activators exert their regulatory function in a more direct fashion and couple the translation of their mt-mRNA to the assembly of the respective OXPHOS complex. Three such direct feedback loops have been identified so far [16]: the translation of the mtDNA encoded complex III subunit Cytb is linked to complex III assembly via the Cbp3–Cbp6 complex [34]; the translation of the mtDNA encoded Cox1 subunit of complex IV is connected to complex IV assembly through Mss51 [35–38]; the translation of the mtDNA encoded Atp6p and Atp8p subunits of complex V is activated by assembly intermediates of the catalytically active F1 oligomer of complex V [39, 40].

In the case of complex IV, the coupling is achieved by the dual role of Mss51 [14, 16, 41]. On one hand, Mss51 binds the 5′ UTR of *COX1* mRNA and acts as a translational activator. On the other hand, Mss51 also interacts with the newly synthesized Cox1 protein in early assembly intermediates of complex IV. Sequestration of Mss51 into this assembly intermediate traps Mss51, hence limiting its availability as translational activator and reducing Cox1 translation. Release of Mss51 from the assembly intermediate only occurs upon progression of complex IV assembly. This Mss51 recycling mechanism therefore matches Cox1 translation to complex IV assembly and prevents the potentially harmful accumulation of non-assembled subunits [42–44]. Intriguingly, it has recently been shown that Mss51 is a heme-binding protein and that heme binding is crucial for the activity of Mss51 [45]. Heme therefore seems to be an important regulator of the coordinated Cox1 translation and assembly into complex IV. In addition to translational activators, also mitochondrial ribosomal proteins can play a role in the translation regulation of specific mtDNA encoded subunits [46, 47]. In the case of the large mitochondrial ribosomal subunit mL38, its regulatory effect on Cox1 translation seems to be dependent on complex IV assembly, hence coupling the two processes as seen for the feedback loops involving the translational activators [48]. The overall importance of translational regulation as control knob to tune mitochondrial gene expression in yeast has also been elegantly demonstrated by shifting yeast cells from glucose, a fermentable carbon source, to glycerol, a non-fermentable carbon source, and thereby shifting energy production towards respiration via OXPHOS [49]. This study not only illustrated that mitochondrial gene expression is shaped by nuclear gene expression, but also that this is orchestrated on the translational level in addition to the transcriptional level in yeast.

Apart from *S. cerevisiae,* also *Saccharomyces pombe* has proven to be a valuable model system to study regulation of mitochondrial translation and its connection to OXPHOS assembly. While the genomic content of the *S. pombe* mitochondrial genome is identical to that of *S. cerevisiae*, hence encoding the same set of proteins, the genome and transcriptome structure as well as overall respiratory physiology more closely resemble the mammalian situation [12, 50, 51]. As such, *S. pombe* presents an interesting intermediate in which the function of certain translational activators present in *S. cerevisiae* has been conserved whereas other translational factors are missing or fullfill only post-translational roles [46, 52].

## Orchestrated mitochondrial translation and OXPHOS assembly in mammals

**Figure 2:**
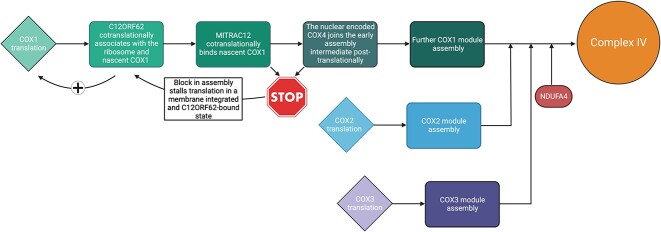
Schematic representation of mammalian complex IV assembly. Assembly of the COX1 module starts with consecutive cotranslational binding of C12ORF62 and MITRAC12 to the COX1 nascent chain, with C12ORF62 additionally associating with the COX1 translating mitochondrial ribosome. Subsequently, COX4 as the first nuclear encoded subunits joins the assembly intermediate posttranslationally. A block in complex IV assembly caused by a lack of MITRAC12 or COX4 results in stalling of the ribosome nascent chain complex in a C12ORF62 bound fashion and therefore a decrease in newly synthesized COX1. After successful progression of further assembly steps of the COX1 module, the COX2 and COX3 module as well as NDUFA4 join to build functional complex IV. The figure has been created using BioRender.com.

The human mtDNA, for example, is a circular genome of about 16.6 kb in size [53]. It is transcribed as two almost genome-length polycistrones which are then further processed to liberate 11 mRNAs (two of which are bicistronic): six mRNAs encoding seven complex I subunits, one mRNA encoding a complex III subunit, three mRNAs encoding complex IV subunits, and one mRNA encoding two complex V subunits [7] (Fig. 1). Unlike yeast mt-mRNAs, mammalian mt-mRNAs possess only very short or no 5′ UTRs, making any regulation via translational activators unlikely. Indeed, the translational activators in yeast do not have functional human homologues. It is therefore not surprising that the regulatory mechanism controlling mtDNA gene expression in mammals largely differ from that in yeast. Transcript-specific translational control is increasingly recognized as an important regulator for mitochondrial gene expression in mammals. For example, in a patient presenting with late-onset Leigh syndrome and complex IV deficiency, mutations in TACO1 have been identified [54]. TACO1 has been shown to specifically bind the *COX1* mRNA and to promote its translation, thereby acting as a mammalian translational activator [54, 55] while its just recently identified yeast counterpart seems to act as a general translational activator [56]. In addition to TACO1, three feedback loops linking translation and OXPHOS assembly have been uncovered in mammals thus far, many more likely still awaiting discovery. One such feedback mechanism connects the early assembly steps of complex IV to COX1 translation through the MITRAC components C12ORF62 or MITRAC12 (Fig. 2). Patients harboring mutations in either *C12ORF62* or *MITRAC12* demonstrated a clear complex IV assembly defect together with a specific reduction of COX1 translation [57, 58]. C12ORF62 and MITRAC12 bind nascent COX1 cotranslationally and in a consecutive order, C12ORF62 additionally engaging selectively with the COX1 translating mitochondrial ribosome [59]. A block of early complex IV assembly, for example through the loss of MITRAC12 or COX4, the first nuclear encoded subunit to join the assembly intermediate, leads to stalling of the ribosome-nascent chain complex in a C12ORF62 bound manner. This consequently leads to a decrease in newly synthesized COX1 and allows for adapting COX1 translation to the efficiency of complex IV assembly. A similar mechanism has also been shown for complex III [60]. Here, patients with mutations in the complex III assembly factor *UQCC2* show complex III deficiency, disturbed complex III assembly, as well as reduced levels of the newly synthesized mtDNA-encoded complex III subunit cytochrome *b*. UQCC2 is required for the stability of complex III assembly factor UQCC1, which in turn binds newly synthesized cytochrome *b*. Finally, also complex I assembly possibly feeds-back on the translation of mtDNA-encoded complex I subunits [61]. Similar to C12ORF62, MITRAC15 associates cotranslationally with the nascent complex I subunit ND2, interacts with the translating ribosome and thereby promotes ND2 translation by a yet unknown mechanism, and is also part of the complex I ND2/P_p_-b module assembly intermediate. While this strongly suggests a link between complex I assembly and translation of ND2, further experiments are needed to clarify the situation and reveal the underlying molecular mechanisms.

## Outlook

Yeast has been a powerful model system to understand the molecular underpinnings of coordinated mitochondrial translation and OXPHOS assembly. In contrast, in mammals we are just starting to understand the orchestrated mitochondrial gene expression. One of the reasons for this discrepancy was the incapability to perform forward genetics in the mammalian system due to the lack of directed mtDNA gene editing methods. Two breakthrough discoveries, namely the development of DddA-derived cytosine base editors (DdCBEs) and the generation of morpholinos, now open up new avenues to modify the mtDNA and translation of individual mt-mRNAs respectively [62, 63]. These tools will allow us to explore and dissect the mechanisms adapting mitochondrial translation to OXPHOS assembly in mammals at an unprecedented depth in the future.
